# Responsiveness to Hedgehog Pathway Inhibitors in T-Cell Acute Lymphoblastic Leukemia Cells Is Highly Dependent on 5′AMP-Activated Kinase Inactivation

**DOI:** 10.3390/ijms22126384

**Published:** 2021-06-15

**Authors:** Valeria Tosello, Deborah Bongiovanni, Ludovica Di Martino, Cinzia Franchin, Paola Zanovello, Giorgio Arrigoni, Erich Piovan

**Affiliations:** 1 UOC Immunologia e Diagnostica Molecolare Oncologica, Istituto Oncologico Veneto IOV—IRCCS, 35128 Padova, Italy; valeria.tosello@iov.veneto.it (V.T.); paola.zanovello@unipd.it (P.Z.); 2 Dipartimento di Scienze Chirurgiche, Oncologiche e Gastroenterologiche, Universita’ di Padova, 35128 Padova, Italy; deborah.bongiovanni@studenti.unipd.it (D.B.); ludovica.dimartino@studenti.unipd.it (L.D.M.); 3 Department of Biomedical Sciences, University of Padova, 35131 Padova, Italy; cinzia.franchin@unipd.it (C.F.); giorgio.arrigoni@unipd.it (G.A.); 4 Proteomics Center, University of Padova and Azienda Ospedaliera di Padova, 35129 Padova, Italy; 5 CRIBI Biotechnology Center, University of Padova, 35131 Padova, Italy

**Keywords:** T-cell lymphoblastic leukemia, hedgehog signaling, 5′AMP-activated kinase signaling, targeted therapy, leukemia growth

## Abstract

Numerous studies have shown that hedgehog inhibitors (iHHs) only partially block the growth of tumor cells, especially in vivo. Leukemia often expands in a nutrient-depleted environment (bone marrow and thymus). In order to identify putative signaling pathways implicated in the adaptive response to metabolically adverse conditions, we executed quantitative phospho-proteomics in T-cell acute lymphoblastic leukemia (T-ALL) cells subjected to nutrient-depleted conditions (serum starvation). We found important modulations of peptides phosphorylated by critical signaling pathways including casein kinase, mammalian target of rapamycin, and 5′AMP-activated kinase (AMPK). Surprisingly, in T-ALL cells, AMPK signaling was the most consistently downregulated pathway under serum-depleted conditions, and this coincided with increased GLI1 expression and sensitivity to iHHs, especially the GLI1/2 inhibitor GANT-61. Increased sensitivity to GANT-61 was also found following genetic inactivation of the catalytic subunit of AMPK (AMPKα1) or pharmacological inhibition of AMPK by Compound C. Additionally, patient-derived xenografts showing high GLI1 expression lacked activated AMPK, suggesting an important role for this signaling pathway in regulating GLI1 protein levels. Further, joint targeting of HH and AMPK signaling pathways in T-ALL cells by GANT-61 and Compound C significantly increased the therapeutic response. Our results suggest that metabolic adaptation that occurs under nutrient starvation in T-ALL cells increases responsiveness to HH pathway inhibitors through an AMPK-dependent mechanism and that joint therapeutic targeting of AMPK signaling and HH signaling could represent a valid therapeutic strategy in rapidly expanding tumors where nutrient availability becomes limiting.

## 1. Introduction

T-cell acute lymphoblastic leukemia (T-ALL) is an aggressive hematological tumor due to the malignant transformation of developing T-cells expressing immature T-cell markers [1]. Notwithstanding intensified polychemotherapy regimens, the outcome of relapsed and chemoresistant T-ALL remains disappointing. Thus, novel and less toxic therapeutic strategies are required that target aberrantly activated signaling pathways. Metabolic homeostasis is a trait that is lost in cancer in order to satisfy the heightened demand for metabolites necessary for growth and proliferation [2]. Although oncogenic mutations can directly alter cellular metabolism in a cell-intrinsic way, microenvironmental cues such as hypoxia, nutrient availability, and crosstalk from neighboring cells all affect cancer metabolism. Leukemia often expands in a hypoglycemic and hypoxic environment (bone marrow, thymus), which contributes to the generation of metabolic heterogeneity within the tumors [3]. Identifying how cancer cells adapt to metabolically adverse conditions and whether this feature of cancer cells can be turned into a vulnerability is a critical issue. Stemness properties have been implicated in cell survival following stress conditions, and they mediate tumor initiation, metastasis, and therapeutic resistance [4,5]. The Hedgehog (HH) pathway is a tightly regulated stemness pathway that is important not only during early development but also for adult tissue maintenance and repair functions [6,7]. HH signaling controls cellular functions by regulating the activities of Glioma-associated oncogene (GLI1-3) transcription factors. Aberrant activation of HH signaling has been identified in a variety of cancer types [8], including T-ALL [9]. AMP-activated protein kinase (AMPK) orchestrates the cellular metabolic state and confers cancer cells with the ability to cope with metabolic stresses [10]. AMPK is a heterotrimeric complex composed of a catalytic α subunit and two regulatory subunits (β and γ), which regulate its activation and substrate specificity [11]. AMPK activation by depleted energy levels, promotes metabolic homeostasis by activating ATP-producing pathways such as glucose uptake, glycolysis, fatty acid uptake and lipid oxidation, and mitochondrial biogenesis while at the same time inactivating ATP-consuming processes such as fatty acid, cholesterol, and protein synthesis [10,12].

Recent studies suggest that AMPK can exert pro- or antitumorigenic roles in cancer, depending on the context [10]. In hematological tumors such as T-ALL, AMPK has been shown to restrain tumor growth [13], and pharmacological activation of AMPK can slow leukemic cell growth through inhibition of mammalian target of rapamycin complex 1 (mTORC1) signaling [14], p38 MAPK [15], or unfolded protein response signaling [16]. On the other hand, oncogenic signals such as RAS, MYC, and NOTCH1 can generate metabolic stress, and AMPK may support cancer cell survival under such conditions [9]. Indeed, in T-ALL, NOTCH1 overexpression is able to induce metabolic stress that leads to AMPK activation that acts to restrain glycolysis through inhibition of mTORC1 and promotes mitochondrial oxidative metabolism to mitigate stress essential for T-ALL survival in vivo [17]. Moreover, AMPK inhibition was shown to enhance apoptosis in MLL-rearranged pediatric B-ALL [18]. Interestingly, AMPK has been reported to directly phosphorylate and destabilize GLI1 protein, inhibiting GLI1 nuclear localization resulting in the suppression of HH signaling [19,20]. Although recent studies have shown that HH signaling is active in a subgroup of T-ALL cases and may play a role in T-ALL progression, only modest therapeutic activity has been found using HH inhibitors (iHHs) in monotherapy [21,22]. Thus, identification of signaling pathways that alter sensitivity to iHHs under metabolic conditions encountered within the tumor microenvironment is imperative for increasing their therapeutic efficacy [23]. In the present report, we analyzed how nutrient starvation (mimicked through serum deprivation) altered iHHs sensitivity in T-ALL cells. 

## 2. Results

### 2.1. Serum Deprivation Increases Responsiveness to GANT-61 and Determines Decreased AMPK Signaling and Increased mTORC1 Signaling 

**Figure 1:**
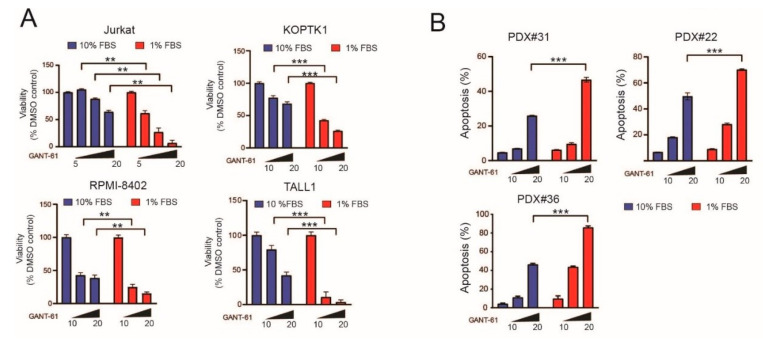

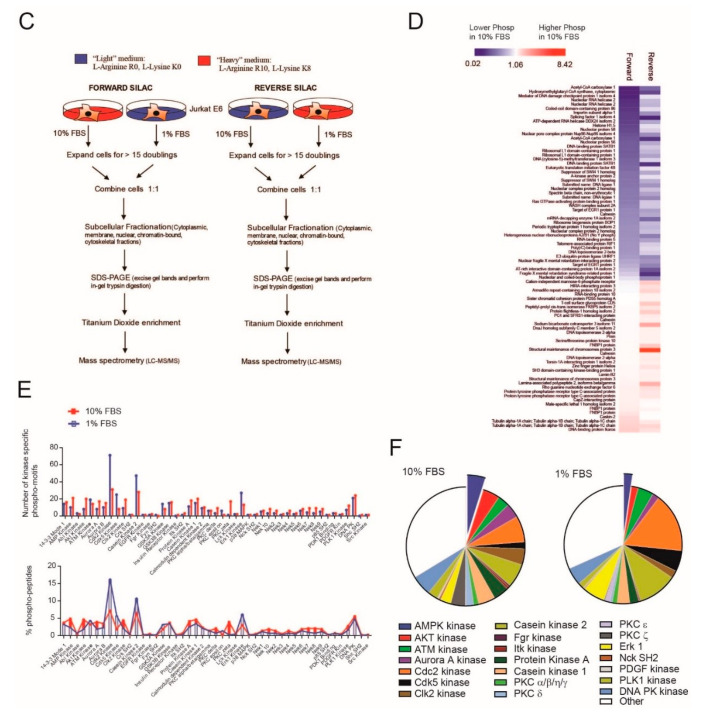
Serum deprivation augments sensitivity to the cytotoxic effects of Hedgehog pathway inhibitors such as GANT-61 through the modulation of numerous signaling pathways. (**A**) Cell viability assays in T-ALL cell lines (Jurkat (24 h), KOPTK1 (48 h), RPMI8402 (48 h), and TALL1 (48 h)) subjected to different culture conditions (10% FBS or 1% FBS) and treated with increasing doses of GANT-61 (5–20 µM). Data are expressed as percentage compared to vehicle (DMSO)-treated control cells (100%). Results of one of three experiments (with similar results) executed in triplicate are shown. Results are shown as the mean ± SD. ** *p* < 0.01, *** *p* < 0.001. (**B**) Apoptosis quantification in patient-derived xenografts (PDX; #22, #31, #36) subjected for 48 h to different culture conditions (10% FBS or 1% FBS) and treated with increasing doses of GANT-61 (10–20 μM). Results of one of two experiments (with similar results) performed in triplicate are shown. Results are shown as the mean ± SD. *** *p* < 0.001. (**C**) Schematic representation of the experimental setup for SILAC experiments. (**D**) Heatmap representation of differentially enriched phosphopeptides (corresponding to specific proteins) following forward and reverse SILAC experiments. (**E**) Graphical representation of kinase specific phospho-motifs (top) or % phosphopeptides (bottom) for a specific kinase present under nutrient-rich (10% FBS) or depleted (1% FBS) conditions. (**F**) Pie charts representing the main kinases active under each experimental condition based on kinase-specific phospho-motifs present on identified phosphopeptides.

Tumor cells undergoing serum starvation in vitro partially mimic metabolically stressed cells trying to adapt to a changed metabolic environment in vivo. This adaptive response modulates signal transduction pathways that could alter responsiveness to iHHs. In certain cell types, such as fibroblasts, increased HH signaling has been reported following nutrient deprivation [24], due to an increased number of ciliated cells. However, primary cilia have not been found in hematopoietic cells [25], and so the effect of serum deprivation on the response to iHHs in T-ALL cells is currently unknown. To address this issue, we compared sensitivity to GANT-61 in T-ALL cells cultured under standard serum conditions (10% FBS) and under serum-deprived (1% FBS) conditions. Serum starvation significantly increased sensitivity to the cytotoxic effects of GANT-61 in all T-ALL cell lines and PDX samples tested (Figure 1A,B and Supplementary Figure S1A). Similar results were obtained for SMO-targeting drugs (cyclopamine and GDC-0449; see Supplementary Figure S1B). To identify the signaling pathways modulated following serum starvation in T-ALL cells, and possibly responsible for the observed change in sensitivity, we performed stable isotope labeling by amino acids in cell culture (SILAC) experiments in which Jurkat T-ALL cells were cultured in light and heavy medium for over 15 cell doublings. The experiment was executed with an isotope labeling “swap” method (see Section 4). To increase the number of identified phosphopeptides, five different cellular fractions (cytoplasmic, membrane, nuclear, chromatin-bound, cytoskeletal) were obtained and analyzed (Supplementary Tables S1 and S2; Figure 1C). Western blot for fractionation-specific markers disclosed that all cellular fractions obtained were highly enriched (Supplementary Figure S1C). The list of phosphopeptides recovered from the different cellular fractions under the two culture conditions (10% FBS vs. 1% FBS) were merged. We found 47 phosphopeptides (corresponding to 39 unique proteins) with a reduced abundance and 36 phosphopeptides (corresponding to 29 unique proteins) with increased abundance under nutrient-rich conditions (10% FBS compared to 1% FBS; Figure 1D,E and Supplementary Figure S1D). Scan site motif software was used to identify putative kinase motifs within these phosphopeptides. Under nutrient-rich conditions (10% FBS), we found phosphopeptides enriched for numerous kinase motifs including, AMPK, DNA-PK, PKA, and AKT (Figure 1E,F). On the other hand, CK2, ATM, ERK, and CDK2/5 kinase motifs were enriched in phosphopeptides found under nutrient-deficient (1% FBS) conditions (Figure 1E,F). 

**Figure 2:**
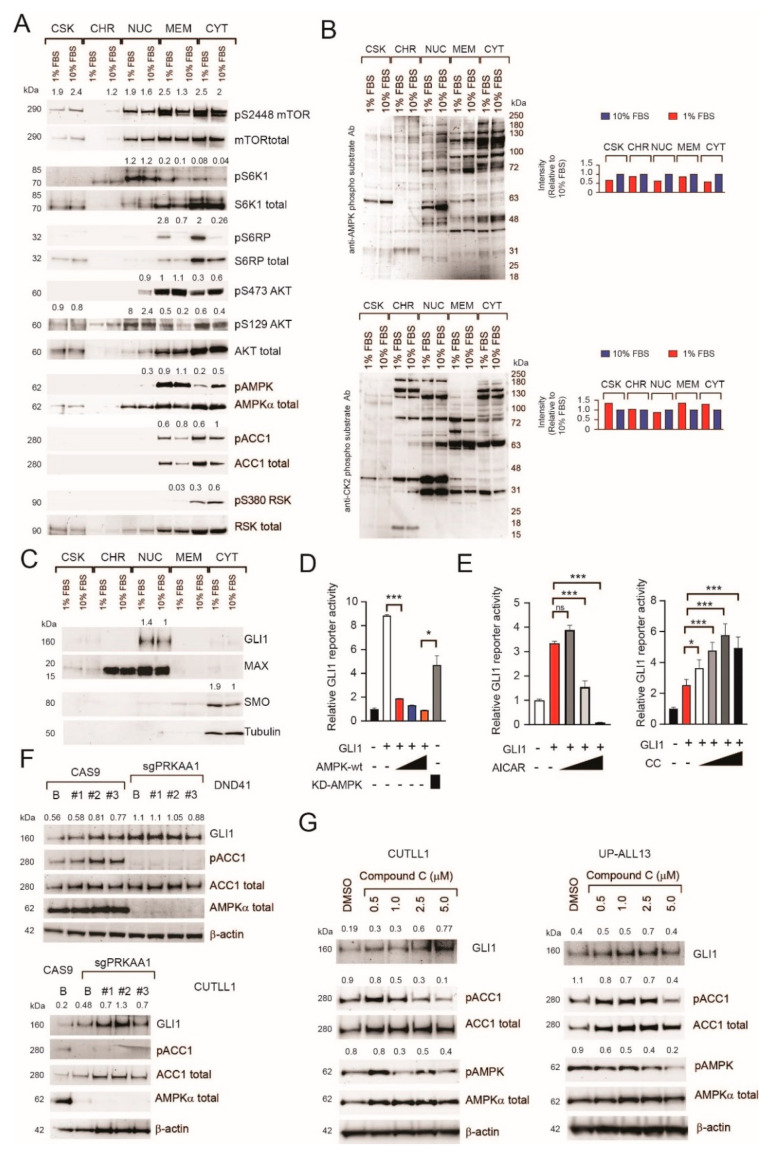
Analysis of signaling pathways modulated following acute nutrient deprivation. (**A**) Cellular fractionation of Jurkat T-ALL cells cultured in nutrient-replete conditions (10% FBS) or nutrient-depleted conditions (1% FBS) for 24 h and evaluated for the activation of specific signaling pathways (PI3K–AKT–mTOR, MAPK–RSK, AMPK, and CK2). Fractions: CSK = cytoskeletal; CHR = chromatin bound; NUC = nuclear; MEM = membrane; CYT = cytoplasmic. The ratio between phosphorylated and total protein is shown for each signaling molecule. (**B**) Cellular fractions of Jurkat T-ALL cells cultured in nutrient-replete conditions (10% FBS) or nutrient-depleted conditions (1% FBS) for 24 h were subjected to immunoblotting and hybridized with anti-phospho AMPK (top) or anti-phospho CK2-substrate (bottom) motif antibodies. (**C**) Cellular fractions of Jurkat T-ALL cells cultured in nutrient-replete conditions (10% FBS) or nutrient-depleted conditions (1% FBS) for 24 h were evaluated for the expression levels of HH pathway components (GLI1 and SMO). Max and tubulin were used as loading controls for nuclear and cytoplasmic fractions, respectively. Normalized GLI1 and SMO protein expression (relative to its loading control) is shown. (**D**) Dual luciferase reporter assay of HEK-293T cells transfected with GLI reporter and GLI1 or empty vector. The effects of increasing amounts of transfected AMPK on GLI reporter were evaluated. Wt = wild type; KD = kinase dead. Results of one of two experiments (with similar results) performed in triplicate are shown. Results are shown as the mean ± SD. * *p* < 0.05, *** *p* < 0.001. (**E**) HEK-293T cells transfected with GLI reporter and GLI1 (or empty vector) were treated with increasing amounts of AICAR (AMPK agonist; 0.5–2 mM; left panel) or Compound C (CC; 1–10 μM; right panel). Relative luciferase activity is represented as fold change relative to empty vector. Results of one of two experiments (with similar results) performed in triplicate are shown. Results are shown as the mean ± SD. Ns = not significant, * *p* < 0.05, *** *p* < 0.001. (**F**) Immunoblot evaluating the expression levels of GLI1, phospho ACC1 (S79), total-ACC1, and AMPKα subunit in AMPKα knockout (sgPRKAA1) or control (CAS9) DND41 (top) or CUTLL1 (bottom) cells in the bulk population (**B**) or clones (#). β-actin was used as a loading control. Normalized GLI1 protein expression (relative to its loading control) is shown. (**G**) Immunoblot evaluating the expression levels of GLI1, phospho ACC1 (S79), total-ACC1, phospho AMPK (T172), and total AMPKα in CUTLL1 (left) or UP-ALL13 (right) cells treated with increasing concentrations of Compound C or vehicle. β-actin was used as a loading control. Normalized GLI1 protein expression (relative to its β-actin loading control) is shown. The ratio between phosphorylated and total protein is shown for ACC1 and AMPKα.

To validate some of the key findings, we executed Western blot analysis for proteins indicative of activation of the following pathways: PI3K–AKT–mTOR (phospho mTOR (S2448), phospho S6K1 (T389), phospho S6 (S235/236), phospho AKT (S473)), MAPK-RSK (phospho RSK (S380)), AMPK (phospho AMPK (T172), phospho ACC (S79)), and CK2 (phos-pho AKT (S129)) in independent cells subjected to cellular fractionation. We found mTORC1 and CK2 substrates to be more highly phosphorylated under serum-deprived conditions (pS6K1, pS6, pmTOR, and pS129 AKT, respectively), whereas AMPK and mTORC2 substrates were more highly phosphorylated under serum-rich conditions (pT172 AMPK and pS473 AKT, respectively), thus validating the SILAC findings (Figure 2A). Results for AMPK and CK2 signaling were further confirmed using phospho AMPK and phospho CK2-substrate motif antibodies (Figure 2B). Given our initial finding, that serum deprivation increases sensitivity to GANT-61 (targeting GLI1/2) and cyclopamine/GDC-0449 (targeting SMO) we also evaluated whether these two proteins were modulated under our experimental conditions. Indeed, we found that serum deprivation modestly increased their expression (GLI1 ≈ 1.4 fold; SMO ≈ 1.9 fold; Figure 2C), possibly contributing to the observed increased therapeutic response.

### 2.2. Genetic Inactivation or Pharmacological Inhibition of AMPK Stabilizes GLI1 Proteins in T-ALL Cells

AMPK has been shown to directly phosphorylate and destabilize GLI1 in brain tumor cells [19,20], whereas mTORC1 activation (through S6K1) has been shown to phosphorylate GLI1 in esophageal cells and increase its nuclear translocation [26]. Further, activated AMPK inhibits the mTORC1 pathway through phosphorylation of tuberous sclerosis 2 or hamartin and raptor [27,28]. Since nutrient deprivation (mimicked by serum starvation) induces inhibition of AMPK and activation of mTORC1 in T-ALL cells, leading to increased response to the cytotoxic effects of GANT-61, we were interested in evaluating the therapeutic importance of targeting AMPK signaling to increase responsiveness to GLI inhibitors. Transfection experiments using a GLI reporter construct in HEK-293T cells confirmed previous reports showing that AMPK can repress the transcriptional activity of GLI1 [19] and that this activity is as least in part due to its catalytic activity (Figure 2D). Additionally, pharmacological activation of AMPK through AICAR [29] determined repression of GLI1 activity, whereas AMPK inhibition by Compound C [30] activated GLI1 transcriptional activity (Figure 2E). 

We next turned to genetic inactivation of AMPK in T-ALL cells to determine its effects on GLI1 protein expression and on response to GLI1 inhibition by GANT-61. To this end, we executed KO experiments by CRISPR–Cas9 technology using sg RNAs targeting the catalytic subunit α1 (AMPKα1/*PRKAA1* gene) almost exclusively expressed in T-cells [31]. The loss of the AMPK α1 subunit resulted in a marked decrease of AMPKα protein expression, along with a reduction in the phosphorylation of AMPK target ACC (Figure 2F). Of note, GLI1 protein levels were modestly increased (≈1.5–2 fold) in AMPK KO T-ALL cells (Figure 2F). Interestingly, the AMPK inhibitor Compound C determined similar results on GLI1 protein levels in T-ALL cells (Figure 2G). The loss of AMPK in T-ALL cells was also associated with increased mTORC1 activity [17] (Supplementary Figure S2). 

### 2.3. A Subgroup of T-ALL Samples Demonstrates an Inverse Relationship between GLI1 Expression Levels and AMPK Signaling Activation

**Figure 3:**
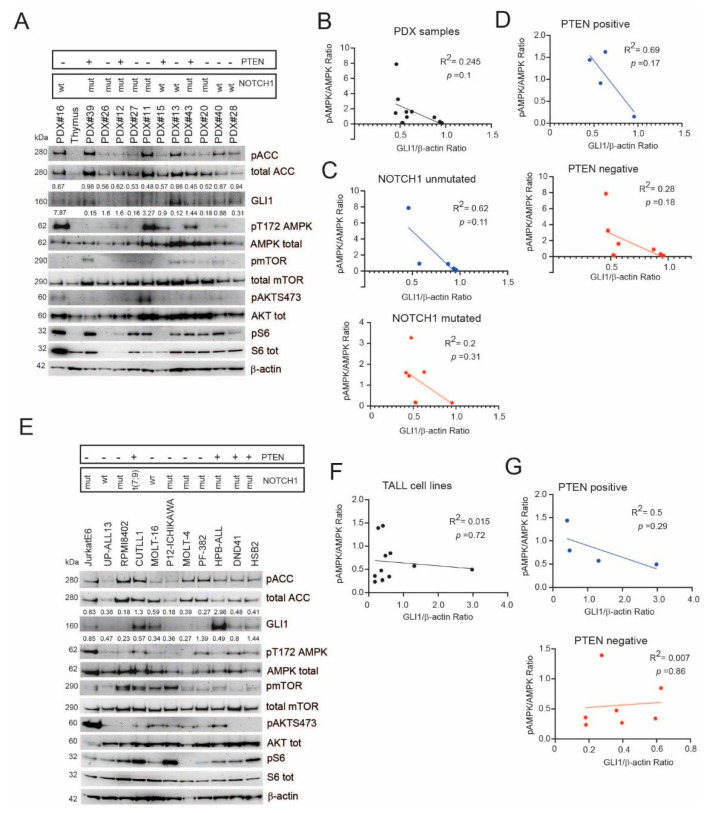
Sustained AMPK activation in a subset of T-ALL cells (PDX samples and cell lines) is associated with low GLI1 expression. (**A**) Western blot evaluating the expression levels in PDX samples of activated AMPK (phospho AMPK (T172)), AMPK targets (phospho ACC1 (S79)), GLI1, mTORC activation (phospho mTOR (S2448), phospho AKT (S473), phospho S6 (S235/236)). Total ACC1, total AMPK, total AKT1/2/3, total S6, and β-actin were used as loading controls. Normalized GLI1 protein expression (relative to its β-actin loading control) is shown. The ratio between phosphorylated and total AMPKα protein is shown. NOTCH1 mutational status and PTEN protein expression status is also reported. NOTCH1 wt = unmutated; NOTCH1 mut = mutated; +, expressed; -, not expressed. (**B**) Linear regression analysis of the relationship between normalized GLI1 protein levels and activated AMPK (pAMPK/AMPKtotal) in all PDX samples. (**C**) Linear regression analysis of the relationship between normalized GLI1 protein levels and activated AMPK (pAMPK/AMPKtotal) in PDX samples divided on the basis of NOTCH1 mutational status. (**D**) Linear regression analysis of the relationship between normalized GLI1 protein levels and activated AMPK (pAMPK/AMPKtotal) in PDX samples divided on the basis of PTEN expression. (**E)** Western blot evaluating the expression levels in T-ALL cell lines of activated AMPK (phospho AMPK (T172)), AMPK targets (phospho ACC1 (S79)), GLI1, mTORC activation (phospho mTOR (S2448), phospho AKT (S473), phospho S6 (S235/236)). Total ACC1, total AMPK, total AKT1/2/3, total S6, and β-actin were used as loading controls. Normalized GLI1 protein expression (relative to its β-actin loading control) is shown. The ratio between phosphorylated and total AMPKα protein is shown. NOTCH1 mutational status and PTEN protein expression status is also reported. NOTCH1 wt = unmutated; NOTCH1 mut = mutated; t(7;9) = translocation generating ICN1; +, expressed; -, not expressed. (**F**) Linear regression analysis of the relationship between normalized GLI1 protein levels and activated AMPK (pAMPK/AMPKtotal) in all T-ALL cell lines. (**G**) Linear regression analysis of the relationship between normalized GLI1 protein levels and activated AMPK (pAMPK/AMPKtotal) in T-ALL cell lines divided on the basis of PTEN expression.

To determine the relevance of our findings, we evaluated the relationship between GLI1 protein expression and AMPK pathway activation in T-ALL cells. We found that PDX samples expressing higher levels of GLI1 tended to have lower levels of activated AMPK (pT172), whereas PDX samples showing higher levels of activated AMPK tended to have lower levels of GLI1 expression (Figure 3A). However, this inverse correlation was not statistically significant (Figure 3B). Subdividing PDX samples according to the presence of NOTCH1-activating mutations or expression of PTEN (two common traits in T-ALL) disclosed that there was a stronger trend for this inverse relationship in NOTCH1 unmutated and PTEN-expressing PDX samples (Figure 3C,D). T-ALL cell lines showed a more complex relationship between GLI1 protein levels and activated AMPK (Figure 3E,F). However, PTEN-expressing T-ALL cell lines also showed a trend for an inverse relationship between GLI1 protein levels and activated AMPK (Figure 3G).

### 2.4. Genetic Inactivation of AMPK Increases Sensitivity to GANT-61 in T-ALL Cells

**Figure 4:**
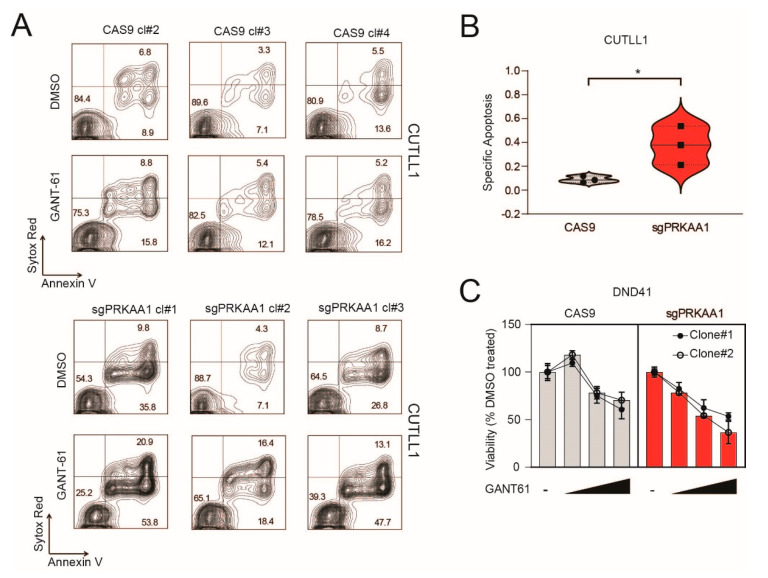

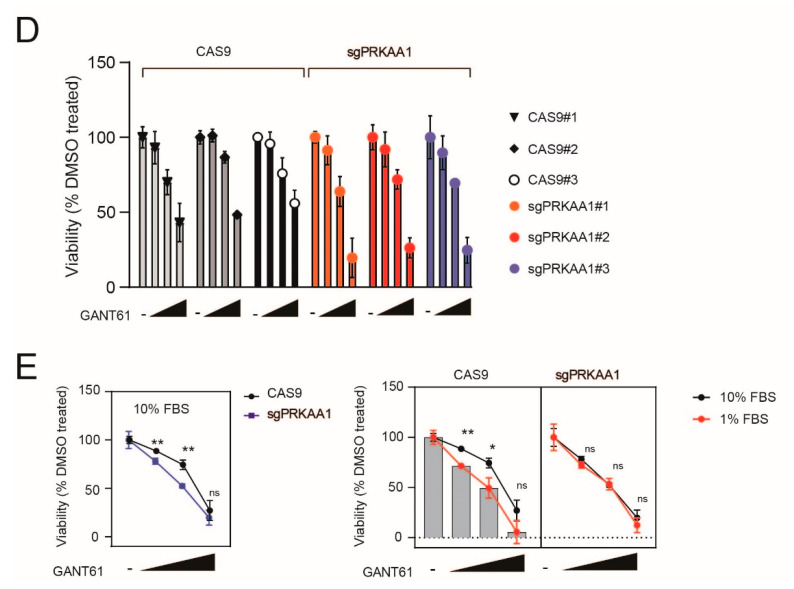
Genetic inactivation of AMPKα increases sensitivity to GANT-61. (**A**) Representative plots of apoptosis in control CAS9 CUTLL1 clones (#2, #3, #4) or AMPKα knockout clones (sgPRKAA1; #1, #2, #3) untreated or treated with GANT-61 (20 µM) for 48 h. (**B**) Quantitation by violin plot of specific apoptosis in control CAS9 CUTLL1 clones or AMPKα knockout clones following exposure to GANT-61 (20 µM) for 48 h. * *p* < 0.05 (**C**) Viability assays of control CAS9 DND41 clones (#1, #2) or AMPKα knockout clones (sgPRKAA1; #1, #2) treated with increasing concentrations of GANT-61 (10–30 µM) for 72 h. Data are expressed as percentage compared to vehicle (DMSO)-treated control cells (100%). Results of one of two experiments (with similar results) performed in triplicate are shown. Results are shown as the mean ± SD. (**D**) Viability assays of control CAS9 Jurkat clones (#1, #2, #3) or AMPKα knockout clones (sgPRKAA1; #1, #2, #3) treated with increasing concentrations of GANT-61 (5–20 µM) for 72 h. Data are expressed as percentage compared to vehicle (DMSO)-treated control cells (100%). Results of one of two experiments (with similar results) performed in triplicate are shown. Results are shown as the mean ± SD. (**E**) Cell viability assays in control CAS9 Jurkat cells or AMPKα knockout cells (sgPRKAA1) subjected to different culture conditions (10% FBS or 1% FBS) and treated with increasing doses of GANT-61 (5–20 µM) for 72 h. Data are expressed as percentage compared to vehicle (DMSO)-treated control cells (100%). Results of one of two experiments (with similar results) performed in quadruplicate are shown. Results are shown as the mean ± SD. Ns = not significant, * *p* < 0.05, ** *p* < 0.01.

We subsequently evaluated whether AMPK-depleted cells had altered sensitivity to the GLI1 inhibitor, GANT-61. We generated CRISPR–Cas9 control and sgPRKAA1 clones in CUTLL1 cells and evaluated their sensitivity to the GLI inhibitor, GANT-61. We found that although AMPK KO clones showed rather variable apoptosis under standard culture conditions, these cells were more sensitive to the cytotoxic effects of GANT-61 (Figure 4A,B). Similar results were obtained in DND41 and Jurkat sgPRKAA1 clones (Figure 4C,D). Interestingly, the increased sensitivity to the cytotoxic effects of GANT-61 in AMPK KO cells was no longer present under serum-depleted conditions (Figure 4E), suggesting that AMPK is a key player in dictating the cytotoxic response to GANT-61 under these conditions.

### 2.5. Pharmacological Inhibition of AMPK Sensitizes T-ALL Cells to the Cytotoxic Effects of GANT-61

**Figure 5:**
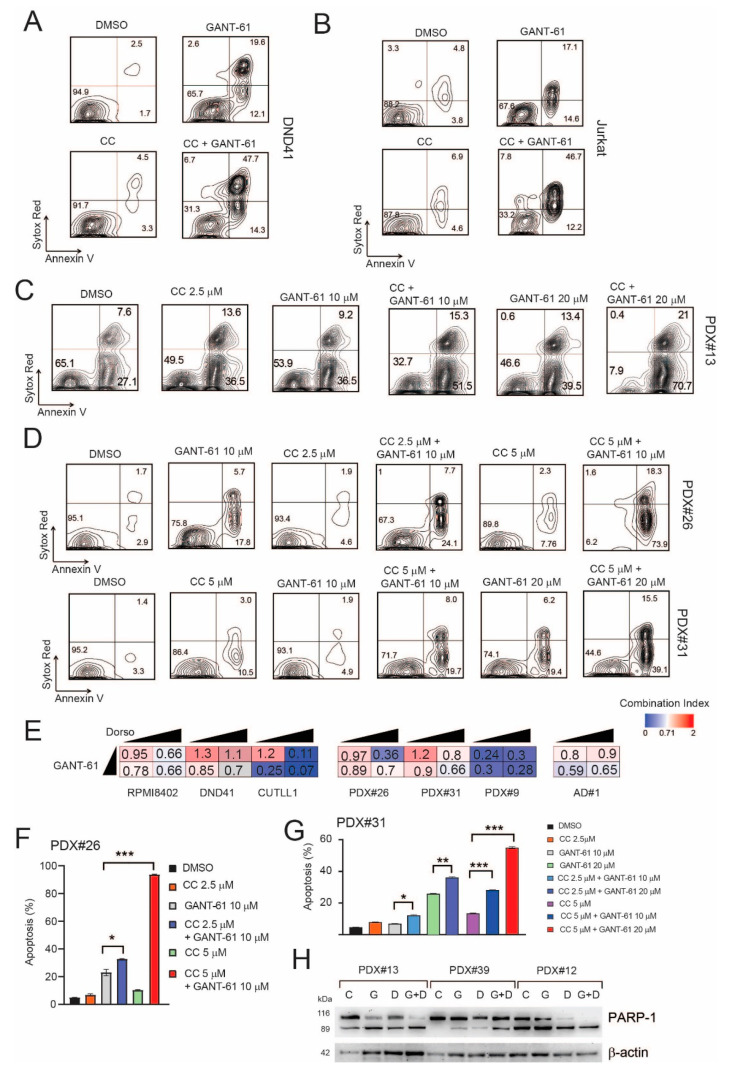
Pharmacological inhibition of AMPK increases the cytotoxic effects of GANT-61 in T-ALL cells. (**A**,**B**) Representative plots of apoptosis in DND41 (**A**) or Jurkat (**B**) T-ALL cells treated in vitro for 48 h with DMSO (vehicle), Compound C (CC) only (2.5 μM), GANT-61 only (20 μM), or CC + GANT-61. (**C**) Representative plots of apoptosis in patient-derived xenograft (PDX#13) T-ALL cells treated in vitro for 48 h with DMSO (vehicle), Compound C (CC) only (2.5 μM), GANT-61 only (10–20 μM, PDX#13), or CC + GANT-61. (**D**) Representative plots of apoptosis in patient-derived xenograft (PDX#26, #31) T-ALL cells treated in vitro for 48 h with DMSO (vehicle), Compound C (CC) only (2.5–5 μM), GANT-61 only (10–20 μM), or CC + GANT-61. (**E**) Heat map representation of combination indexes between GANT-61 and Compound C (CC) in T-ALL cell lines (RPMI8402, DND41, CUTLL1), patient-derived xenografts (PDX#26, #31, #9), and patient-derived leukemic cells (AD#1). GANT-61 was used at 10 or 20 µM, while Compound C was used at 2.5 or 5 µM. CI > 1.1 indicates antagonism, CI < 1 indicates synergism. (**F**) Quantification of apoptosis in PDX#26 treated in vitro for 48 h with DMSO (vehicle), Compound C (CC) only (2.5 or 5 μM), GANT-61 only (10 or 20 μM), or combinations of CC + GANT-61. Results of one of two experiments (with similar results) performed in triplicate are shown. Results are shown as the mean ±SD. * *p* < 0.05, *** *p* < 0.001. (**G**) Quantification of apoptosis in PDX#31 treated in vitro for 48 h with DMSO (vehicle), Compound C (CC) only (2.5 or 5 μM), GANT-61 only (10 or 20 μM), or combinations of CC + GANT-61. Results of one of three experiments (with similar results) performed in triplicate are shown. Results are shown as the mean ±SD. * *p* < 0.05, ** *p* < 0.01, *** *p* < 0.001. (**H**) Western blot evaluating the expression levels of PARP-1 in PDX samples (#13, #39, #12) treated in vitro for 48 h with DMSO (vehicle; C), Compound C (D) only (2.5 or 5 μM), GANT-61 only (G; 10 or 20 μM), or combinations of CC + GANT-61 (G + D). β-actin was used as loading control.

Next, we tested the antileukemic effects of jointly targeting the AMPK and Hedgehog signaling pathways in T-ALL. To this end, we treated numerous T-ALL cell lines (CUTLL1, DND41, Jurkat, RPMI-8402), PDX samples (#26, #31, #9 and #13), and a primary T-ALL sample (AD#1) in vitro with vehicle, GANT-61, Compound C, or the combination (GANT-61 + Compound C). These experiments demonstrated that there was an additive/synergistic antileukemic effect between the AMPK inhibitor (Compound C) and GANT-61 in all T-ALL cell lines and PDX samples tested (Figure 5A–G and Supplementary Figure S3). As several SMO inhibitors are in clinical development for the treatment of cancer, we also evaluated whether targeting AMPK signaling would increase the therapeutic efficacy of these drugs. We found T-ALL cells to be rather resistant to the SMO drugs tested (cyclopamine and GDC-0449) with only very high doses (>10–20 µM for cyclopamine or >40 µM GDC-0449) determining any effects on cell viability/proliferation (Supplementary Figure S1B). Notwithstanding this limit, there was a modest increase in the antiproliferative effects of GDC-0449 when combined with the AMPK inhibitor (Compound C) in some T-ALL cell lines (Supplementary Figure S4A). Further, we also tested a more recent AMPK inhibitor, SBI-0206965 [32,33] in combination with GANT-61. Again, we found that SBI-0206965 was able to increase the cytotoxic effects of GANT-61 in the T-ALL cells tested (Supplementary Figure S4B–E). Conversely, AMPK activation through metformin did not increase the cytotoxic effects of GANT-61 in Jurkat T-ALL cells (Supplementary Figure S4F,G).

## 3. Discussion

Cancer cells residing in metabolically adverse conditions, such as those with low oxygen, glucose, and nutrient levels, need to adapt metabolically in order to maintain an energetic balance and continue to proliferate. The elucidation of signaling pathways responsible for this response could expose new therapeutic vulnerabilities. Stemness properties have been implicated in cell survival against stress conditions, and they mediate tumor initiation, metastasis, and therapeutic resistance [4,5]. HH signaling is a stemness pathway crucial during embryonic development but largely inactive in adult life except during tissue repair [7]. However, aberrant activation of HH signaling has been identified in a variety of cancer types [34] (including T-ALL [21,22,35]), driving proliferation, self-renewal, and tumorigenesis. Extensive crosstalk exists between the HH pathway and other oncogenic or stemness signaling pathways, such as RAS/RAF/MERK/ERK, PI3K/AKT, mTOR/S6K1, EGFR, and NOTCH1 [36]. In T-ALL, a subgroup of cases presents evidence of active HH signaling and iHHs (especially GLI1/2 targeting GANT-61) partially block the growth of human T-ALL cells in vitro and in vivo [21]. It is becoming clear that the metabolic milieu of the tumor microenvironment influences the behavior of tumors. Leukemia cells in different locations may encounter different levels of metabolic stress, including low nutrient availability, which is partially mimicked through serum starvation. In lung carcinoma cells, HH signaling is augmented under stress conditions such as serum starvation to maintain survival, proliferation, and self-renewal [24]. We find that serum starvation of T-ALL cells significantly increased responsiveness to HH pathway inhibitors such as GANT-61 and cyclopamine/GDC-0449 in vitro. We performed quantitative phosphoproteomics (SILAC) in Jurkat T-ALL cells to identify the main signaling pathways that become modulated following serum starvation. We found enrichment in phosphopeptides containing putative motifs for known metabolic regulators such as kinases of the PI3K/AKT/mTOR and AMPK signaling pathways. Surprisingly, acute nutrient deprivation resulted in suppression of AMPK signaling and activation of mTORC1 signaling, suggesting leukemia cells may adapt to this stressful condition by activating mTORC1 signaling. The mechanism/s behind this activation is/are currently unknown; however, BCAT1 enzyme induction following serum starvation (data not shown), implicated in branched chain amino acid (BCAA) metabolism [37], may be implicated in this process. Our results imply that serum deprivation increases responsiveness to GLI1/2 targeting agents in T-ALL cells possibly through increased stability of GLI1 protein determined by augmented S6K1-mediated S84 phosphorylation [26] and decreased AMPK-dependent S408 (and possibly S104, S1074) phosphorylation [19,20]. Given the numerous studies evaluating the role of mTORC1 signaling and its therapeutic targeting in T-ALL [9,38], we focused our research on the role of AMPK signaling in T-ALL and its crosstalk with HH signaling. 

The alterations seen in serum-deprived T-ALL cells (AMPK and mTOR signaling components) are reminiscent of those seen following AMPK deficiency. Indeed, AMPKα1 KO T-ALL cells showed increased mTORC1 activity. Consistently, AMPKα1 KO cells showed increased GLI1 protein expression and increased sensitivity to GANT-61 in vitro. Our findings may have therapeutic relevance, as inhibition of AMPK by Compound C sensitizes leukemia cells to the cytotoxic effects of the GLI1/2 inhibitor GANT-61 in vitro. However, it should be noted that in vitro screening has found that Compound C is rather promiscuous, inhibiting multiple kinases [39]. Thus, the results obtained in this study will need to be confirmed using more-specific AMPK inhibitors. As a step toward this aim, we evaluated the effects of SBI-0206965 (which inhibits the ULK1–AMPK axis). This drug demonstrated similar effects to those of Compound C when combined with GANT-61, while it was ineffective when combined with the SMO inhibitor GDC-0449 (data not shown). This suggests that AMPK signaling may be more important for regulating sensitivity to GLI1/2 inhibitors rather than to SMO inhibitors. 

It is becoming clear that AMPK may exert tumor-suppressing and tumor-promoting effects, depending on the context [10]. Numerous factors may influence the outcome of AMPK signaling including degree/mechanisms of AMPK activation, AMPK isoform expression, AMPK subcellular localization, activity of other signaling pathways in the cell, and microenvironmental conditions. In T-ALL, it seems that AMPK signaling is frequently activated, possibly due to oncogenic NOTCH1 signaling [17], where it promotes cell survival through increased oxidative metabolism. Further, PI3K/AKT/mTOR is also frequently activated in T-ALL samples [40,41]. Thus, at least in T-ALL, there may be an attenuation of the classical inhibitory effect of AMPK signaling on mTORC1 through the phosphorylation of tuberous sclerosis 2 or hamartin and raptor. Given our results, one could speculate that when resources (nutrients) are limited, T-ALL cells may actually downregulate the activity of AMPK, which no longer restrains mTORC1 signaling and activates HH signaling, thus favoring tumor growth and invasive behavior rather than acting as a tumor suppressor. On the other hand, under nutrient-replete conditions, AMPK activation may be important to mitigate metabolic stress through the promotion of metabolic plasticity. Thus, both AMPK activation (through metformin/phenformin) and AMPK inhibition (Compound C/SBI-0206965 derivatives [33]) could be useful in combination treatment regimens for T-ALL patients. In the present study, we demonstrate a synergistic therapeutic effect of jointly targeting the HH and AMPK signaling pathways in vitro in T-ALL models. Future studies executed in vivo using clinical-grade iHHs (targeting GLI transcription factors) and specific AMPK pathway inhibitors will be required to determine the utility of the proposed combination treatment.

## 4. Materials and Methods

### 4.1. Cell Culture and Stable Isotope Labeling 

Heavy lysine and arginine ([^13^C_6_,^15^N_2_]-L-lysine and [^13^C_6_,^15^N_4_]-L-arginine) were purchased from Cambridge Isotope Laboratories (Andover, MA, USA). RPMI SILAC medium was purchased from Thermo Fisher Scientific (Waltham, MA, USA). All other chemicals were purchased from Sigma-Aldrich (Merck, Darmstadt, Germany) and were of high purity or MS grade if not otherwise specified. For SILAC experiments, Jurkat T-ALL cells were cultured in RPMI medium containing either heavy lysine and arginine (heavy cells) or conventional L-lysine and L-arginine (light cells), supplemented with 1 mM L-glutamine, 1% penicillin/streptomycin, and 10% dialyzed fetal bovine serum (FBS; Gibco, Thermo Fisher Scientific). Cells were subjected to serum-deprivation experiments after 15 cell passages to ensure complete labeling of proteins. Two biological replicates were obtained with a tag-swapping strategy. For forward SILAC experiments, 50 × 10^6^ Jurkat T-ALL cells grown in RPMI 10% FBS “heavy” medium (heavy cells) were mixed with 50 × 10^6^ Jurkat T-ALL cells grown in RPMI 1% FBS “light” medium (light cells) for 24 h (ratio 1:1; Mix A) before being subjected to cellular fractionation. In reverse SILAC experiments, 50 × 10^6^ Jurkat T-ALL cells grown in RPMI 10% FBS “light” medium (light cells) were mixed with 50 × 10^6^ Jurkat T-ALL cells grown in RPMI 1% FBS “heavy” medium (heavy cells) for 24 h (ratio 1:1; Mix B) before being subjected to cellular fractionation. The subcellular protein fractionation kit (Thermo Fisher Scientific) was used to extract cytoplasmic, membrane, nuclear-soluble, chromatin-bound, and cytoskeletal protein fractions, following the manufacturer’s instructions. Each subcellular fraction was then quantified using the BCA method (Pierce, Pero, Italy). Forward and reverse SILAC subcellular fractions (250 µg each) were denatured and loaded onto 4–12% SDS-PAGE precast gels (NuPAGE Bis-Tris Gel, Invitrogen, Carlsbad, CA, USA) and separated at 150 V for 1 h. Gels were stained with SimplyBlue Coomassie (Invitrogen) and destained overnight in water. Each gel lane was subjected to in-gel protein digestion [42]. In detail, proteins were in-gel reduced with 10 mM dithiothreitol (DTT) in 50 mM NH_4_HCO_3_ at 56 °C for 1 h and alkylated with 55 mM iodoacetamide in 50 mM NH_4_HCO_3_ at room temperature for 45 min in the dark. Next, 400 µL of 12.5 ng/μL sequencing-grade modified trypsin (Promega, Madison, WI, USA) in 50 mM NH_4_HCO_3_ was added to each sample and protein digestion was carried on overnight at 37 °C. Peptides were extracted from the gel by three consecutive treatments with 50% acetonitrile (ACN)/0.1 formic acid (FA), dried under vacuum, and stored at −20 °C.

### 4.2. Phosphopeptide Enrichment 

Phosphopeptide enrichment was performed with homemade microcolumns prepared by inserting 500 μg of TiO_2_ (Titansphere, GL Sciences Inc., Tokyo, Japan) into Stage Tips (C18 material, Thermo Fisher Scientific). Each peptide sample (Mix A and Mix B) was suspended in 150 µL of loading/washing buffer (80% ACN/6% trifluoroacetic acid (TFA)). Columns were conditioned twice with ACN (50 µL) and twice with loading buffer (50 µL). Samples were slowly loaded into the microcolumns, and the resin was washed twice with 50 µL of the following solutions: 50% ACN/6% TFA, 200 mM NaCl, washing buffer, and 0.1% TFA. Retained peptides were eluted with 100 µL of 10% NH_4_OH freshly prepared (pH ≈ 11.0) and collected in a vial containing 10 µL of FA. A second elution from the C_18_ filter was performed with 100 µL of 50% ACN/0.1% FA. Samples were dried under vacuum and suspended in 40 μL of 3% ACN/0.1% FA for LC–MS/MS analysis.

### 4.3. LC-MS/MS and Data Analysis

LC–MS/MS analyses were performed with an LTQ-Orbitrap XL mass spectrometer (Thermo Fisher Scientific) coupled with a nano-HPLC Ultimate 3000 (Dionex, Thermo Fisher Scientific). Phosphopeptides were loaded into a homemade pico-frit column (75 μm I.D., 15 μm Tip, New Objective) packed with C_18_ material (Aeris peptide 3.6 μm XB-C18, Phenomenex) and separated using a linear gradient of ACN/0.1% FA (from 3% to 50% in 90 min), at a flow rate of 250 nL/min. The instrument operated in a data-dependent mode; the ion source capillary temperature was set at 200 °C and the spray voltage was optimized at 1.3 kV. To increase the number of identified peptides and the confidence in phosphosite localization, each sample was analyzed three times with different fragmentation methods (MS^2^, neutral loss triggered MS^3^, and MS^2^ with multistage activation) [43]. Raw files were analyzed with the software Proteome Discoverer 1.4 (Thermo Fisher Scientific) connected to a Mascot server (version 2.2.4, Matrix Science Ltd., London, UK). Data were searched against the human section of the UniProt database (version 2015.04.01, 90,411 entries). Enzyme specificity was set to trypsin with up to three missed cleavages. Mass tolerance was set to 10 ppm for parent mass and to 0.6 Da for fragment ions. Carbamidomethylation of cysteine residues was set as fixed modification. L-Arginine-^13^C_6_,^15^N_4_ and L-lysine-^13^C_6_,^15^N_2_ were set as variable modifications, together with methionine oxidation and phosphorylation of serine, threonine, and tyrosine residues. Data were analyzed with a MudPIT protocol, by merging (for each of the experimental replicates) all data obtained from the three technical replicates (MS^2^, MS^3^, and multistage activation). The algorithm Percolator was used to assess the reliability of peptide identifications and filter the results: only peptides with a false discovery rate (FDR) <0.01 were considered as positive hits. Only unique peptides were considered for quantification, which was performed directly using the Proteome Discoverer software. For each peptide, the final quantification value was obtained as the average value of all replicates (technical and experimental). Only phosphopeptides that were quantified in at least one of the replicates from Mix A and one from Mix B were retained for further analysis. To increase the robustness of the quantification, all peptides that showed a discordant trend across replicates (due to incomplete labeling, arginine to proline conversion, or inconsistent quantification) were discarded. For each phosphopeptide, modulations identified by the “forward” and “reverse” technique were averaged. Furthermore, we considered log2 fold changes of −0.5 (heavy to light (H/L) ratios <0.7) and 0.5 (H/L ratios >1.4) as biological threshold levels for significant phosphoprotein modulations. Thus, phosphopeptides had to show significant modulation (log2 (H/L) ≤−0.5 or ≥0.5) at least in one condition, and this modulation had to be consistent between “forward” and “reverse” experiments. We used Scansite 4.0 to search for motifs within isolated peptides that are likely to be phosphorylated by specific protein kinases.

### 4.4. Cell Lines and In Vitro Treatments 

T-ALL cell lines (CUTLL1, DND41, JURKAT E6, RPMI-8402, TALL1, KOPTK1) were cultured in complete RPMI-1640 medium (EuroClone, Pero, Italy) supplemented with 10% FBS at 37 °C under 5% CO_2_. UP-ALL13 cells [44] were cultured in complete RPMI-1640 medium supplemented with 20% FBS. HEK-293T were cultured in complete DMEM medium (EuroClone), supplemented with 10% FBS. Primary leukemia cells (AD#1) were obtained from the peripheral blood (PB) of a male patient at diagnosis. Informed consent and approval by the Azienda Ospedaliera di Padova Review Board were obtained according to general guidelines, conforming with the Declaration of Helsinki.

### 4.5. T-ALL Xenografts 

Patient-derived xenografts (PDX) have been previously established and described [45]. T-ALL PDX cells were expanded in vivo via intravenous injection in NOD *Rag1*^null^*IL2Rγ*^null^ (NSG, Jackson Laboratory, Bar Harbor, ME, USA) immunodeficient mice. For in vitro studies, T-ALL cells from xenografted mice were cultured in MEM-α medium supplemented with 10% human serum and cytokines for 48 h. Procedures involving animals and their care conformed with institutional guidelines. The study was approved by the Institutional Review Board (OPBA) of University of Padova (protocol code 82/2015; 16 December 2015) and the Italian Ministry of Health (DGSAF; 618/2016-PR). Tumor-bearing mice were euthanized, and leukemic cells extracted from spleens were used in functional assays.

### 4.6. Cell Viability and Flow Cytometry Analysis 

For in vitro studies, T-ALL cells were plated at a density of 3 × 10^5^/mL in triplicate for each experimental condition in 24-well plates. Cell lines were seeded either in complete RPMI medium supplemented with 1% or 10% FBS, while PDX cells were seeded in complete MEM-α medium (see above) with 1% or 10% human serum. Cell viability was assessed by ATPlite Luminescence ATP Detection Assay System (PerkinElmer, Waltham, MA, USA) after treatment for 24–48 h (serum deprivation experiments) or 48–72 h (drug combination experiments). Viability data are expressed as percentage compared to vehicle (DMSO)-treated control cells (set as 100%). For cell viability and apoptosis assays, cells were treated with GANT-61 (5–30 μM, Selleck Chemicals LLC, Houston, TX, USA) and Compound C (CC: 2.5–5 µM), alone or in combination. Apoptosis was evaluated by flow cytometry following cell staining with Annexin-V-FLUOS Staining Kit (Roche, Basel, Switzerland) and SYTOX Red Dead Cell Stain (Thermo Fisher Scientific) after 48–72 h. Apoptosis was defined as the sum of the percentage of Annexin V^+^/SYTOX Red^−^ and Annexin V^+/^SYTOX Red^+^ cells. Samples were analyzed on a FACSCalibur flow cytometer (BD Biosciences, Franklin Lakes, NJ, USA) supporting Cell Quest software (BD Biosciences), and data were analyzed with FlowJo™ Software (FlowJo LLC, Ashland, OR, USA). The specific apoptosis was calculated as previously described [46].

### 4.7. Plasmids and Lentiviral Production

Inactivation of human AMPKα1 (*PRKAA1* gene) in T-ALL cells using the CRISPR–Cas9 technology was achieved as previously described [47,48]. LentiCRISPRV2 vector was obtained from Addgene. For target sequence (sgRNA) design, we used the following link: http://www.e-cripsr.org/E-CRIPR/deaigncrispr.htlm, accessed on 28 April 2016. AMPKα1 knockout (KO) was done using LentiCRISPRV2 expressing an sgRNA sequence that targets Exon1 of the *PRKAA1* gene (sgRNA: CACCGGCGTGTCACCCAGAATGTAG). For viral production, LentiCRISPRV2 vectors were transfected in HEK-293T cells using JetPEI transfection reagent (Polyplus, Illkirch, France) together with packaging plasmids. The viral supernatant was collected 48 h after transfection, filtered, and used to infect target cells. All infections of T-ALL cells were performed by spinoculation. After infection, T-ALL cells were selected for 5–7 days in puromycin before functional assays and, where possible, limiting dilution cloning (for CRISPR/Cas9 infected cells).

### 4.8. Immunoblotting 

Whole-cell-lysate extraction and Western blot analysis were performed as previously described [49]. For Western blotting, protein samples were separated on 4–12% gradient Tris-Glycine or 3–8% Tris-Acetate SDS-PAGE Gels (Invitrogen) and transferred to a PVDF membrane (Millipore). The primary antibodies used are listed in the “Supplementary Materials and Methods section”. Images were acquired on a ChemiDoc XRS Imager (Bio-Rad, Hercules, CA, USA) acquisition imagine system and analyzed with QuantityOne^®^ 1-D analysis software (Bio-Rad) and ImageJ software (National Institutes of Health, Bethesda, MD, USA). We quantified each protein band using ImageJ software and normalized each target protein after background subtraction to its loading control or to its total protein form (for phosphorylated proteins).

### 4.9. Dual Luciferase Reporter Assays

To study GLI1 transcriptional activity, HEK-293T cells were co-transfected with 8 × 3’Gli-BS-delta51-LucII Firefly luciferase reporter plasmid (a gift from Hiroshi Sasaki [50]; Riken plasmid RDB08061) and pcDNA3.1-GLI1-FLAG (a gift from Prof. Gianluca Canettieri, University La Sapienza, Rome, Italy) expression plasmid. pGL4.74 [hRluc/TK] Renilla luciferase reporter plasmid (Promega) was used for internal normalization of transfection efficiency. In some experiments, AMPK expression vectors (pCIP-AMPKα1_WT and pCIP-AMPKα1_KD, both from Addgene) [51] were also co-transfected. The cell culture medium was switched to serum-deprived DMEM (<2% dialyzed FBS) when cells reached confluence. When AMPK activity was modified pharmacologically (AICAR/Compound C), these drugs were added 24 h post-transfection. Luciferase activity was measured 72 h post-transfection using the Dual-Luciferase Reporter assay kit (Promega). Relative luciferase activity was calculated as Firefly luciferase activity normalized against Renilla luciferase activity.

### 4.10. Statistical Analysis

Results were expressed as mean value ± standard deviation (SD). Student’s t-test and nonparametric *t*-test (Mann–Whitney) were used where appropriate. All statistical tests were two sided and unpaired, and *p* < 0.05 was considered statistically significant (* *p* < 0.05, ** *p* < 0.01, *** *p* < 0.001). Statistical analyses were performed with GraphPad Prism software (GraphPad Software, San Diego, CA, USA). To determine the synergistic, additive, or antagonistic effect of drug combinations, we calculated combination index (CI) values as defined by Chou [52], with CalcuSyn software (Biosoft, Cambridge, UK).

## Data Availability

The data presented in this study are available on request from the corresponding author.

## Supplementary Materials

The following are available online at https://www.mdpi.com/article/10.3390/ijms22126384/s1. Supplementary Figure S1: Serum deprivation increases response to GANT-61 and is associated with modulation of the phosphorylation status of diverse proteins, Supplementary Figure S2: Genetic inactivation of AMPKα in T-ALL cells is associated with reduced phosphorylation of RAPTOR and mTORC2 target S473 AKT1 but increased phosphorylation of mTORC1 targets, Supplementary Figure S3: Pharmacological modulation of AMPK alters the cytotoxic effects of GANT-61 in T-ALL models, Supplementary Figure S4: Pharmacological inhibition of AMPK alters the cytotoxic effects of GANT-61 and GDC-0449 in T-ALL cells, Table S1: Differentially expressed phosphopeptides in forward SILAC experiment (MIX A), Table S2: Differentially expressed phosphopeptides in reverse SILAC experiment (MIX B), Supplementary Materials and Methods: Antibodies used for Immunoblotting.
