# Supplementary material for: Responsiveness to Hedgehog Pathway Inhibitors in T-Cell Acute Lymphoblastic Leukemia Cells Is Highly Dependent on 5′AMP-Activated Kinase Inactivation
Source: Int J Mol Sci. 2021 Jun 15;22(12):6384. doi: 10.3390/ijms22126384 (PMC8232330; doi:10.3390/ijms22126384)
Supplement: Supplementary file 1 [file ijms-22-06384-s001.zip › Supplementary Information.pdf]

International Journal of *Molecular Sciences*

MDPI

Supplementary Information

Responsiveness to Hedgehog Pathway Inhibitors in T-Cell Acute Lymphoblastic Leukemia Cells Is Highly Dependent on 5′AMP-Activated Kinase Inactivation

Valeria Tosello, Deborah Bongiovanni, Ludovica Di Martino, Cinzia Franchin, Paola Zanovello, Giorgio Arrigoni and Erich Piovan *

-Supplementary Figure S1
-Supplementary Figure S2
-Supplementary Figure S3
-Supplementary Figure S4
-Supplementary Table S1
-Supplementary Table S2
-Supplementary Materials and Methods

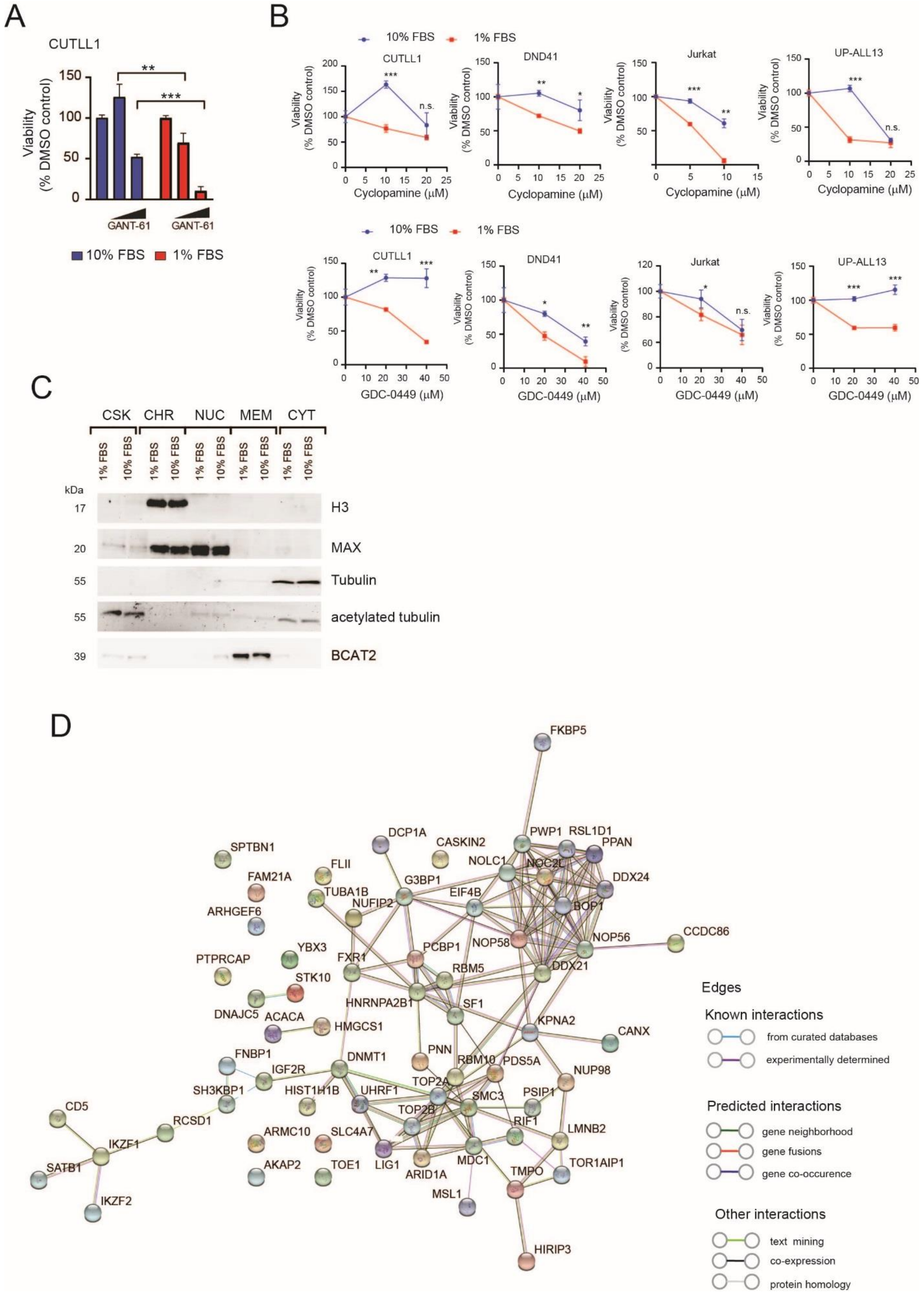

Supplementary Figure S1. Serum deprivation increases response to GANT-61 and is associated with modulation of the phosphoryla tion status of diverse proteins. (**A**) Cell viability assays in CUTLL1 T-ALL cells subjected to different culture conditions (10% FBS or

1% FBS) and treated with increasing doses of GANT-61 (10-20 μM) for 48 h. Data is expressed as percentage compared to vehicle (DMSO) treated control cells (100%). Results of one of three experiments (with similar results) performed in triplicate are shown. Results are shown as the mean ±SD. ** p <0.01, *** p <0.001. (**B**) Viability assays of CUTLL1, DND41, Jurkat and UP-ALL13 T-ALL cells subjected to different culture conditions (10% FBS or 1% FBS) and treated with increasing concentrations of Cyclopamine (top; 5-20 μM) or GDC-0449 (bottom; 20-40 μM) for 72 h. Data is expressed as percentage compared to vehicle (DMSO) treated control cells (100%). Results of one of two experiments (with similar results) performed in triplicate are shown. Results are shown as the mean ±SD. * p <0.05, ** p <0.01, *** p <0.001. (**C**) Cellular fractionation of Jurkat T-ALL cells cultured in nutrient replete conditions (10% FBS) or nutrient deplete conditions (1% FBS) for 24 h and evaluated for fractionation specific markers. The following fractiona tion markers were used: α-tubulin for CYT fraction; BCAT2 for MEM fraction; MAX for NUC fraction; Histone H3 for CHR fraction; acetylated tubulin for CSK fraction. Fractions: CSK= Cytoskeletal; CHR= chromatin bound; NUC= nuclear; MEM=membrane; CYT=cytoplasmic. (**D**) STRING software was used to determine connections between proteins differentially phosphorylated under our experimental conditions (10% FBS vs 1% FBS) identified by mass spectrometry (Confidence view). Network nodes represent proteins, while edges represent protein-protein associations. Stronger associations are represented by thicker lines.

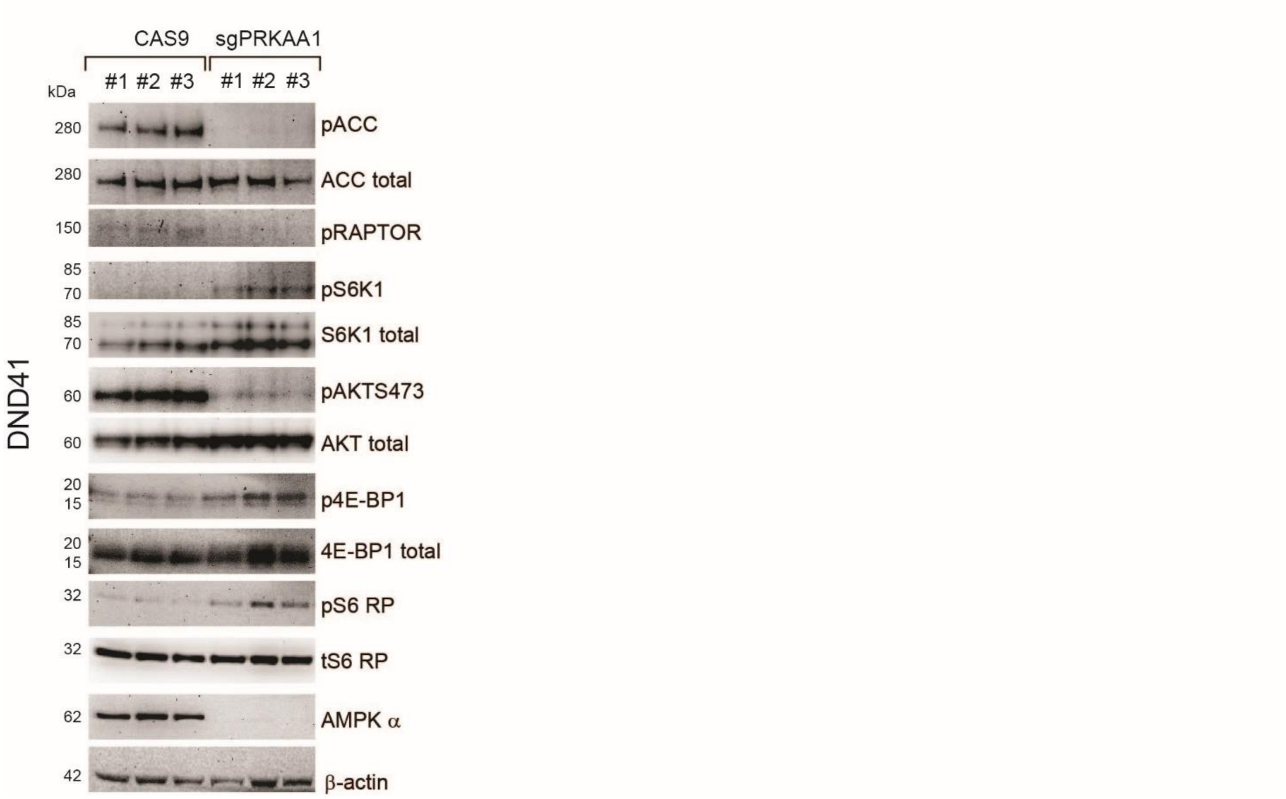

Supplementary Figure S2. Genetic inactivation of AMPKα in T-ALL cells is associated with reduced phosphorylation of RAPTOR and mTORC2 target S473 AKT1 but increased phosphorylation of mTORC1 targets. Western blot evaluating the expression levels of phospho ACC1 (S79), phospho RAPTOR (S792), phospho S6K1 (T396), phospho AKT (S473), phospho 4E-BP1 (S65), phospho S6 (S235/236). Total AMPKα, total -ACC1, total S6K1, total AKT1/2/3, total S6, total 4E-BP1 and β-actin were used as loading controls.

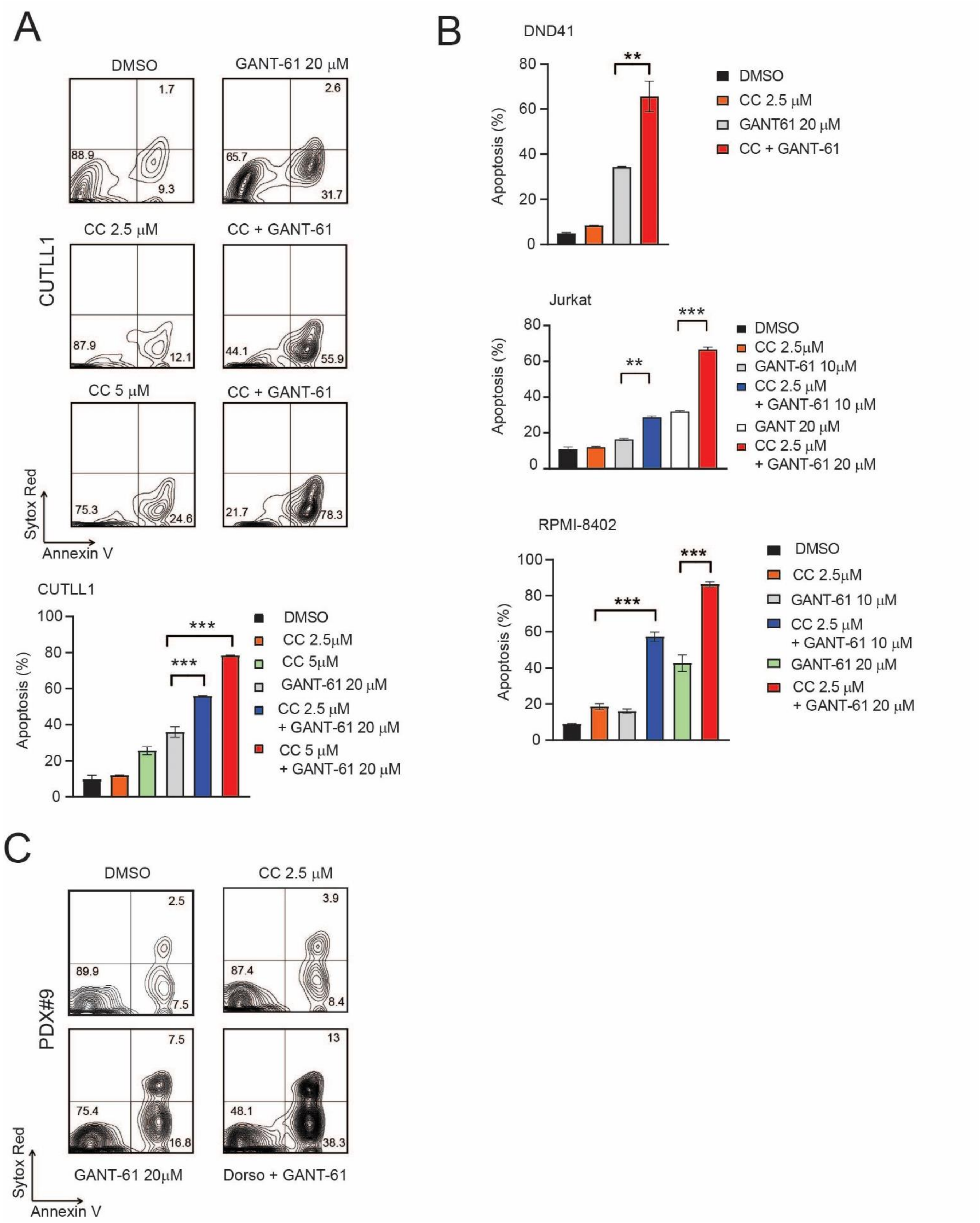

Supplementary Figure S3. Pharmacological modulation of AMPK alters the cytotoxic effects of GANT-61 in T-ALL models. (**A**) Representative plots of apoptosis (top) or quantification of apoptosis (bottom) in CUTLL1 cells treated *in vitro* for 48 h with DMSO (vehicle), Compound C (CC) only (2.5 or 5 μM), GANT-61 only (20 μM) or CC + GANT-61. Results of one of three experiments (with similar results) performed in triplicate are shown. Results are shown as the mean ±SD. *** p <0.001. (**B**) Quantification of apoptosis in

DND41 (top), Jurkat (middle), RPMI8402 (bottom) T-ALL cells treated *in vitro* for 48 h with DMSO (vehicle), Compound C (CC) only (2.5 μM), GANT-61 only (10 or 20 μM) or combinations of CC + GANT-61. Results of one of two experiments (with similar results) performed in triplicate are shown. Results are shown as the mean ±SD. ** $p < 0.01$, *** $p < 0.001$. (**C**) Representative plots of apoptosis in patient derived xenograft (PDX#9) T-ALL cells treated *in vitro* for 72 h with DMSO (vehicle), Compound C (CC) only (2.5 μM), GANT-61 only (20 μM) or CC+ GANT-61.

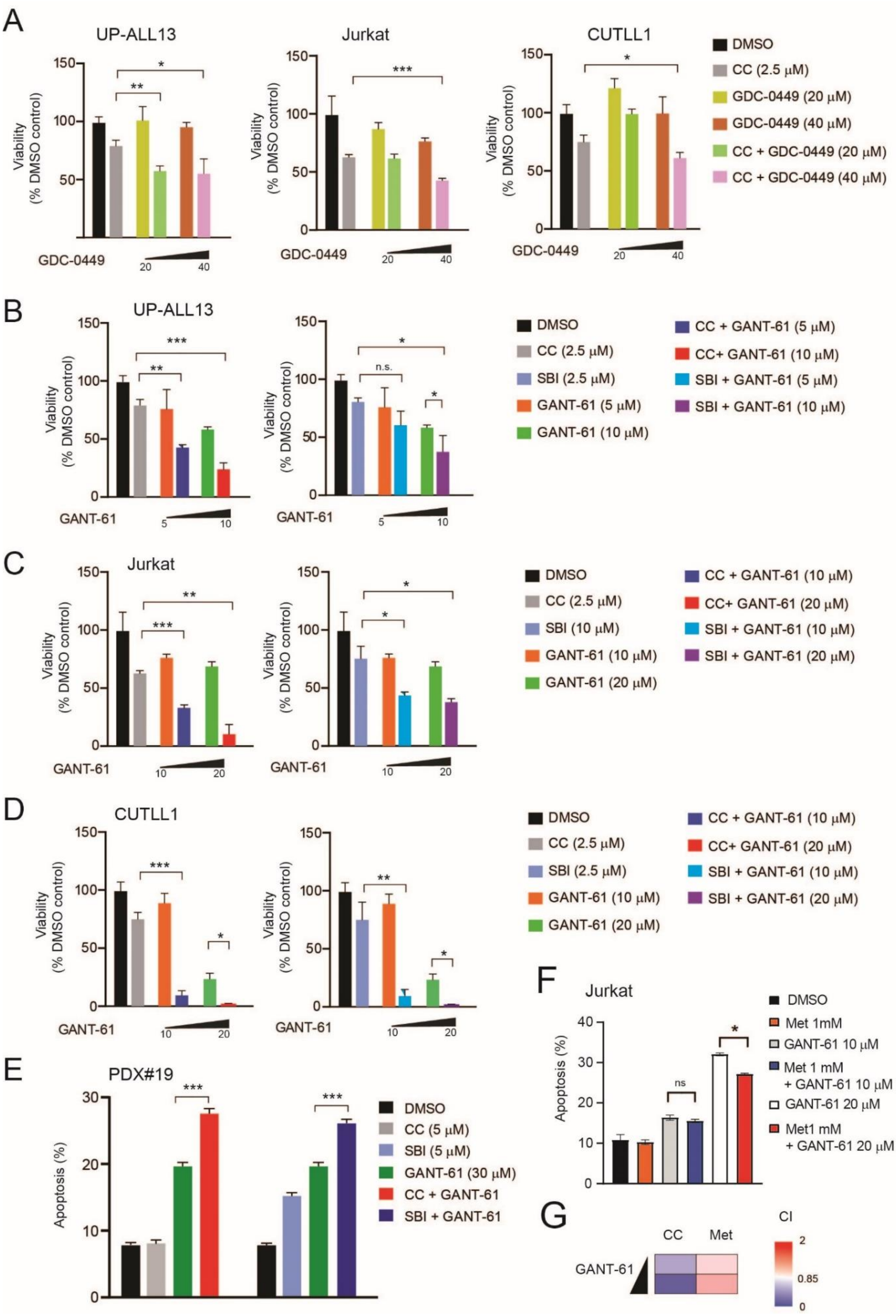

Supplementary Figure S4. Pharmacological inhibition of AMPK alters the cytotoxic effects of GANT-61 and GDC-0449 in T-ALL cells. (**A**) Viability assays of UP-ALL13, Jurkat and CUTLL1 T-ALL cells treated for 72 h with DMSO (vehicle), Compound C (CC)

only (2.5 μM), GDC-0449 only (20-40 μM) or CC + GDC-0449. Data is expressed as percentage viability compared to vehicle (DMSO) treated control cells (100%). Results of one of two experiments (with similar results) performed in triplicate are shown. Results are shown as the mean ±SD. * p <0.05, ** p <0.01, *** p <0.001.(**B**) Viability assays of UP-ALL13 cells treated for 72 h with DMSO (vehicle), Compound C (CC) only (2.5 μM), SBI-0206965 only (SBI; 2.5 μM), GANT-61 only (5-10 μM), CC + GANT-61 or SBI + GANT-61. Data is expressed as percentage viability compared to vehicle (DMSO) treated control cells (100%). Results of one of two experiments (with similar results) performed in quadruplicate are shown. Results are shown as the mean ±SD. * p <0.05, ** p <0.01, *** p <0.001. (**C**) Viability assays of Jurkat cells treated for 72 h with DMSO (vehicle), Compound C (CC) only (2.5 μM), SBI-0206965 only (SBI; 10 μM), GANT-61 only (10-20 μM), CC + GANT-61 or SBI + GANT-61. Data is expressed as percentage viability compared to vehicle (DMSO) treated control cells (100%). Results of one of two experiments (with similar results) performed in triplicate are shown. Results are shown as the mean ±SD. * p <0.05, ** p <0.01, *** p <0.001. (**D**) Viability assays of CUTLL1 cells treated for 72 h with DMSO (vehicle), Compound C (CC) only (2.5 μM), SBI-0206965 only (SBI; 2.5 μM), GANT-61 only (10-20 μM), CC + GANT-61 or SBI + GANT-61. Data is expressed as percentage viability compared to vehicle (DMSO) treated control cells (100%). Results of one of two experiments (with similar results) performed in quadruplicate are shown. Results are shown as the mean ±SD. * p <0.05, ** p <0.01, *** p <0.001. Quantification of apoptosis in PDX#19 cells treated *in vitro* for 72 h with DMSO (vehicle), Compound C (CC; 5 μM) only, SBI-0206965 only (SBI; 5 μM), GANT-61 only (30 μM) or combinations of CC + GANT-61 or SBI + GANT-61. Results of one of three experiments (with similar results) performed in triplicate are shown. Results are shown as the mean ±SD. *** p <0.001. (**F**) Quantification of apoptosis in Jurkat T-ALL cells treated *in vitro* with vehicle (DMSO), GANT-61 (10 or 20 μM), Metformin (1 mM) or the combination (GANT-61+Metformin). Results are shown as the mean ±SD. ns= not significant, * p<0.05. (**G**) Heat map representation of combination indexes at 72 h between GANT-61 and Metformin (Met) or Compound C (CC) in Jurkat T-ALL cells. GANT-61 was used at 10 or 20 μM, Compound C at 2.5 μM and Metformin at 1 mM. CI>1.1 indicates antagonism, CI<1 indicates synergism.

Supplementary Table S1. Differentially expressed phosphopeptides in forward SILAC experiment (MIX A).

Supplementary Table S2. Differentially expressed phosphopeptides in reverse SILAC experiment (MIX B).

Supplementary Materials and Methods:

Antibodies used for Immunoblotting

The following primary antibodies were used for immunoprobing: rabbit anti-GLI1 (68H3, #3538, Cell Signaling Technology, Danvers, MA, USA), rabbit anti-phospho mTOR (Ser2448, #2971, Cell Signaling), rabbit anti-total mTOR (7C10, #2983, Cell Signaling), rabbit anti-phospho-p70 S6 Kinase (Thr389, 108D2, #9234, Cell Signaling), rabbit anti- p70 S6 Kinase (49D7, #2708, Cell Signaling), rabbit anti-phospho-S6 Ribosomal Protein (Ser235/236, D57.2.2E, #4858, Cell Signaling), mouse anti-S6 Ribosomal Protein (54D2, #2317, Cell Signaling), rabbit anti-phospho-Akt (Ser473, D9E, #4060, Cell Signaling), rabbit anti-phospho-4E-BP1 (Ser65,#9456, Cell Signaling), rabbit anti-4E-BP1 (#9452, Cell Signaling),rab-bit anti-phospho-Akt1 (Ser129, D4P7F, #13461, Cell Signaling), rabbit anti-phospho-AMPK Substrate Motif (LXRXX(pS/pT), #5759, Cell signaling), rabbit anti-phospho-CK2 Substrate ((pS/pT) DXE, #8738, Cell Signaling), mouse anti-pan Akt (40D4, #2920, Cell Signaling), rabbit anti-phospho-AMPKα (Thr172, 40H9, #2535, Cell Signaling), rabbit anti-AMPKα (D5A2, #5831, Cell Signaling), rabbit anti-phospho-Acetyl-CoA Carboxylase (Ser79, D7D11, #11818, Cell Signaling), rabbit anti-Acetyl-CoA Carboxylase (C83B10, #3676, Cell Signaling), rabbit anti- phospho-p90RSK (Ser380, D3H11, #11989, Cell Signaling), rabbit anti-RSK1/RSK2/RSK3 (32D7, #9355, Cell signaling), rabbit anti-MAX (C17, sc-197; Santa Cruz Biotechnology, Dallas, TX, USA), mouse anti-tubulin α (TU-02, sc-8035, Santa Cruz Biotechnology), rabbit anti-β-actin (D6A8, #8457, Cell Signaling), rabbit anti-Smo was from LifeSpan BioSciences (Seattle, WA, USA); mouse anti-acetylated tubulin α (6-11B-1, sc-23950, Santa Cruz Biotechnology).
